# Liver fibrosis quantified by image morphometry predicts clinical outcomes in patients with non-alcoholic fatty liver disease

**DOI:** 10.1007/s12072-023-10564-3

**Published:** 2023-06-26

**Authors:** Zhengyi Wang, Gary P. Jeffrey, Yi Huang, Bastiaan De Boer, George Garas, Michael Wallace, Luis Bertot, Leon A. Adams

**Affiliations:** 1 Medical School, The University of Western Australia, Perth, Australia; 2 Department of Hepatology, Sir Charles Gairdner Hospital, Perth, Australia; 3 PathWest Laboratory Medicine, Perth, Australia

**Keywords:** Non-alcoholic steatohepatitis, Long-term clinical outcomes, Collagen proportionate area, Liver-related death, Liver decompensation, Hepatocellular carcinoma, Non-invasive fibrosis tests, Sirius red staining, Hepascore, FIB-4, APRI

## Abstract

**Background and aims:**

Liver fibrosis predicts adverse clinical outcomes, such as liver-related death (LRD) and hepatocellular carcinoma (HCC) in patients with non-alcoholic fatty liver disease (NAFLD). We aimed to investigate the accuracy of semi-automated quantification of collagen proportionate area (CPA) as an objective new method for predicting clinical outcomes.

**Method:**

Liver biopsies from patients with NAFLD underwent computerized image morphometry of Sirius Red staining with CPA quantification performed by ImageScope. Clinical outcomes, including total mortality, LRD, and combined liver outcomes (liver decompensation, HCC, or LRD), were determined by medical records and population-based data-linkage. The accuracy of CPA for predicting outcomes was compared with non-invasive fibrosis tests (Hepascore, FIB-4, APRI).

**Results:**

A total of 295 patients (mean age 50 years) were followed for a median (range) of 9 (0.2–25) years totalling 3253 person-years. Patients with CPA ≥ 10% had significantly higher risks for total death [hazard ratio (HR): 5.0 (1.9–13.2)], LRD [19.0 (2.0–182.0)], and combined liver outcomes [15.6 (3.1–78.6)]. CPA and pathologist fibrosis staging (FS) showed similar accuracy (AUROC) for the prediction of total death (0.68 vs. 0.70), LRD (0.72 vs. 0.77) and combined liver outcomes (0.75 vs. 0.78). Non-invasive serum markers Hepascore, APRI, and FIB-4 reached higher AUROC; however, they were not statistically significant compared to that of CPA except for Hepascore in predicting total mortality (0.86 vs. 0.68, *p* = 0.009).

**Conclusion:**

Liver fibrosis quantified by CPA analysis was significantly associated with clinical outcomes including total mortality, LRD, and HCC. CPA achieved similar accuracy in predicting outcomes compared to pathologist fibrosis staging and non-invasive serum markers.

**Supplementary Information:**

The online version contains supplementary material available at 10.1007/s12072-023-10564-3.

## Introduction

Non-alcoholic fatty liver disease (NAFLD) is a common hepatic manifestation of metabolic disturbances related to excess adiposity and insulin resistance. It affects 1 in 4 adults worldwide and approximately 1 in 5 of these develop non-alcoholic steatohepatitis (NASH) characterized by liver injury and fibrosis [1]. Progression of NASH leads to decompensated cirrhosis, hepatocellular carcinoma (HCC), and liver-related death (LRD). Fibrosis severity in NAFLD is the strongest histological predictor of HCC, liver-related, and total mortality risk [2]. More recently, non-invasive fibrosis tests (NITs) have also been utilized as predictors of histological fibrosis and long-term liver-related outcomes [3–5].

Currently, histological fibrosis is evaluated by immunohistochemical staining, such as Masson’s Trichrome staining of collagen deposits, and staged using a semi-quantitative histological scoring systems which categorize fibrosis from 0 to 4 [6, 7]. Although pathologist staging includes architectural assessment as well as fibrosis quantification, this approach has several potential limitations. First, Masson trichrome may stain other extracellular matrix components and thus may be less specific for collagen [8] whereas Sirius Red fixes higher ordered collagen fibrils found in more mature fibrosis and is more specific [9]. Pathologist interpretation of fibrosis staging is also prone to inter and intra-observer variability [10]. Finally, the semi-quantitative staging system focuses more on the architectural patterns of fibrosis rather than the absolute amount of collagen deposition and is insensitive to small changes in fibrosis which may be clinically relevant or useful in determining response in clinical trial settings. Semi-automated image scanning of Sirius red collagen staining offers the potential to overcome some of these limitations. The ratio of collagen stained area against total scanned area [= collagen proportionate area (CPA)] is used to calculate the fibrosis in each sample. CPA has been suggested as a better predictor of clinical outcomes than histopathological staging in patients after liver transplantation [11]. Our previous studies have also demonstrated among patients with chronic hepatitis C that CPA provides more accurate prediction of clinical outcomes compared to the traditional pathologist staging [12, 13]. Therefore, in this study, we aimed to investigate the accuracy of the collagen proportionate area (CPA) as an objective and reproducible method of predicting adverse clinical outcomes in patients with NAFLD and compared its predictive accuracy with pathologist fibrosis staging and non-invasive fibrosis tests.

## Methods

### Participants

Study participants were patients who attended the Hepatology Department, Sir Charles Gairdner Hospital (Perth, Australia) from 1992 to 2016 with NAFLD and underwent liver biopsy. Biopsies were requested by the treating hepatologist according to their normal practice to assess the severity of liver disease in patients with high liver enzymes or elevated non-invasive fibrosis measures, or to confirm diagnosis and aetiology of liver disease. Initially, 581 patients were identified with further exclusion of patients with a second etiology of chronic liver disease (hepatitis B and C, drug induced liver disease and alcohol excess; *n* = 22), a clinical event prior to liver biopsy (*n* = 8), unusable liver tissue for Sirius Red staining (*n* = 14), or no available clinical records for follow-up (*n* = 90). A cohort of 295 participants were included in the final analysis. The detailed exclusion process is described in Supplementary Fig. 1.

### Clinical and biochemical data collection

Clinical and laboratory data were collected in a standardized fashion from electronic hospital records and pathology records. Demographic features included age, gender, and ethnicity. BMI was calculated using the formula: weight (kilograms)/height^2^ (meters). All biochemical assays including liver function tests, fasting lipids, glucose, and insulin were conducted by the State referral laboratory (Pathwest, Nedlands, WA). Non-invasive markers, including Hepascore, FIB-4, and APRI, were calculated at baseline according to published algorithms [4, 14, 15].

### Clinical outcomes collection

Clinical outcomes were collected based on medical records and population-based data-linkage. The primary end point was liver-related death (LRD; death from liver failure or a liver-related complication, HCC, or transplantation). Secondary outcomes included total mortality (including liver transplantation), liver decompensation (defined by ascites, hepatic encephalopathy, variceal bleeding, hepatorenal syndrome, or spontaneous bacterial peritonitis), and development of HCC. HCC was diagnosed according to standard diagnostic criteria guidelines [16, 17]. Combined liver outcome was defined as liver decompensation, HCC or LRD, whichever occurred first. Clinical outcomes were determined by two methods; first, a comprehensive review of the patient medical record, laboratory, pathology, and radiology records with outcomes confirmed by experienced hepatologists. Second, by ICD-10 codes from the Western Australian Data Linkage Unit (WADLU) which is a validated population-based data-linkage system including registries for cancer, hospital admissions, and mortality for the state of WA. Codes for liver-related death included K72.1, I98.3, and C22.0; codes for liver decompensation included R18, K72.1, I98.3, K76.7, L65, and C22. The WADLU has 100% coverage of data for all hospital admissions and deaths in Western Australia, providing information on principal and additional diagnoses for each episode of care and has been widely used in cohort and population-based studies [18, 19]. There is 98% linkage for Hospital Morbidity Data, 99.5% linkage for the Death Registry, and 99.4% linkage for the Cancer Registry. These are core datasets within the WADLU and are continuously subject to standard linkage quality checks.

### Liver biopsy assessment

Liver histology was reviewed by an experienced histopathologist (B.dB) from the Department of Anatomical Pathology, Pathwest Laboratory (Nedlands, WA). Fibrosis was staged according to the NASH-CRN scoring system. Briefly, fibrosis was classified on a 5-point scale: stage 0 = no fibrosis, stage 1 = zone 3 perisinusoidal/perivenular fibrosis, stage 2 = zone 3 and periportal fibrosis, stage 3 = septal/bridging fibrosis, and stage 4 = cirrhosis.

### Collagen proportionate area determination

Liver biopsy samples were cut to 4 µm slides from stored formalin-fixed paraffin-embedded tissue blocks for Sirius Red Staining which was performed by Pathwest. Briefly, slides were immersed in 0.4 mM Picro-sirius red solution for an hour after being washed by distilled water. Slides were then sealed with coverslips followed by washing in 0.5% acetic acid for 3 min twice. Dried slides were scanned by Aperio ScanScope XT (Leica biosystems, Wetzlar, Germany) under 20 × magnification. Computerized image morphometry was performed by Aperio ImageScope v12.3.3 (Leica biosystems, Wetzlar, Germany). Image analysis was performed by one operator (Z.W.) as illustrated in Supplementary Fig. 2. Briefly, the pen tool was used to outline the whole lvier tissue area. Liver capsule, extra-hepatic tissue, and highly fragmented tissues were then counter-selected and excluded from further analysis (Supplementary Fig. 2d and e). Analysis parameters were set as follows: hue value = 0.95; hue width = 0.3; color saturation threshold = 0.04; Iwp (High) = 255; Iwp (low)—Ip (High) = 175; Isp (low) = 0; Inp (High) = − 1. Parameter Ip (Low) = Isp (High) were tuned between 110 and 175 until brown areas (strongly positive) represented the positive collagen staining, the blue areas (negative) represented all the blank portions (including macrocellular steatosis and ballooning), and the orange and yellow areas (medium and weakly positive) covered the remaining tissue. All other parameters were kept the same for all samples except for Ip (Low) = Isp (High). CPA was calculated as (number of strong positive pixels/number of total pixels) × 100%. The intra-observer variability was tested in 20 random samples and the absolute mean difference in CPA was 0.11 ± 0.04% with the Pearson correlation co-efficient being 0.99. We have previously demonstrated excellent reliability (intra-class co-efficient 0.92) for CPA quantification between batches of immunohistochemical staining [13]. CPA stratification was determined according to a previous study [12]. Group 1 was categorized as CPA < 3%; group 2 CPA 3–5%; group 3 CPA > 5–10%; and group 4 CPA > 10%.

### Statistical analysis

Statistical analyses were performed by IBM^®^ SPSS^®^ Statistics v 26.0 (IBM Corporation, NY, US). Baseline data were presented as mean and standard deviation (SD) or number and percentages as appropriate. Cox regression analysis was used to demonstrate the association between CPA values and risks of long-term clinical outcomes. Confounding factors, including age, diabetes, and hypertension, were adjusted in multi-variate Cox regression models. Area under the receiver-operating characteristic curve (AUROC) was used to demonstrate the predictive accuracy of CPA and NIT markers. A *p* value less than 0.05 was considered as statistically significant.

## Results

### Baseline characteristics

Baseline characteristics of the 295 participants are listed in Table 1. The mean age was 50 ± 13 years with approximately half being female (161/295; 56%). Metabolic disorders were prevalent in the cohort with hypertension and type 2 diabetes diagnosed in 40% of the cohort (111/273; 112/274, respectively), whereas 35.1% of participants were taking medication for dyslipidemia (95/271).

**Table 1:** Baseline characteristics

Variables	Values	*N*
Age, years	50 ± 13	295
Male, *n* (%)	134 (45.4)	295
Race, Caucasian, *n* (%)	115 (98.6)	176
Weight, kg	102 ± 27	190
Height, cm	168 ± 11	157
BMI, kg/m^2^	37.3 ± 9.2	156
Waist, cm	128 ± 89	112
Hip, cm	130 ± 14	42
SBP, mmHg	132 ± 17	188
DBP, mmHg	80 ± 11	188
Diabetes, *n* (%)	112 (40.9)	274
Hypertension, *n* (%)	111 (40.7)	273
Dyslipidaemia, *n* (%)	95 (35.1)	271
Bilirubin, µmol/L	14.7 ± 26.3	275
ALP, IU	102 ± 53	192
ALT, IU	78 ± 82	273
AST, IU	47 ± 41	220
GGT, IU	155 ± 259	264
Alb, g/L	43 ± 6	268
PLT, 10^9^/L	240 ± 72	259
INR	1.4 ± 4.0	131
Creatinine, µmol/L	79 ± 37	171
Glucose, mmol/L	6.4 ± 2.5	218
Insulin, µIU/l	23.1 ± 48.8	95
Cholesterol, mmol/L	4.9 ± 1.5	114
LDL-c, mmol/L	2.9 ± 1.9	105
HDL-c, mmol/L	1.12 ± 0.33	190
Triglycerides, mmol/L	2.9 ± 2.4	206
Hepascore	0.39 ± 0.34	197
APRI	0.60 ± 0.80	207
FIB-4	1.59 ± 2.22	207
Liver histology NAS scoring, *n* (%)		
Steatosis, 0	2 (0.7)	295
1	104 (35.3)	
2	120 (40.7)	
3	69 (23.4)	
Inflammation, 0	104 (35.3)	295
1	164 (55.6)	
2	27 (9.2)	
3	0	
Ballooning, 0	192 (65.1)	295
1	79 (26.8)	
2	24 (8.1)	
Fibrosis, 0	137 (46.4)	295
1	79 (27.0)	
2	27 (9.2)	
3	34 (11.6)	
4	18 (6.1)	

Mean ± standard deviation or number (percentage) as appropriate

Liver biopsy scoring was performed in all participants. Two biopsies without steatosis had established cirrhosis with previous imaging evidence of steatosis in the presence of metabolic risk factors. Most patients had no fibrosis (137/295, 46.4%), whereas 34 (11.6%) demonstrated advanced fibrosis and 18 (6.1%) cirrhosis (Table 1).

### CPA values and correlation with fibrosis stage

**Fig. 1:**
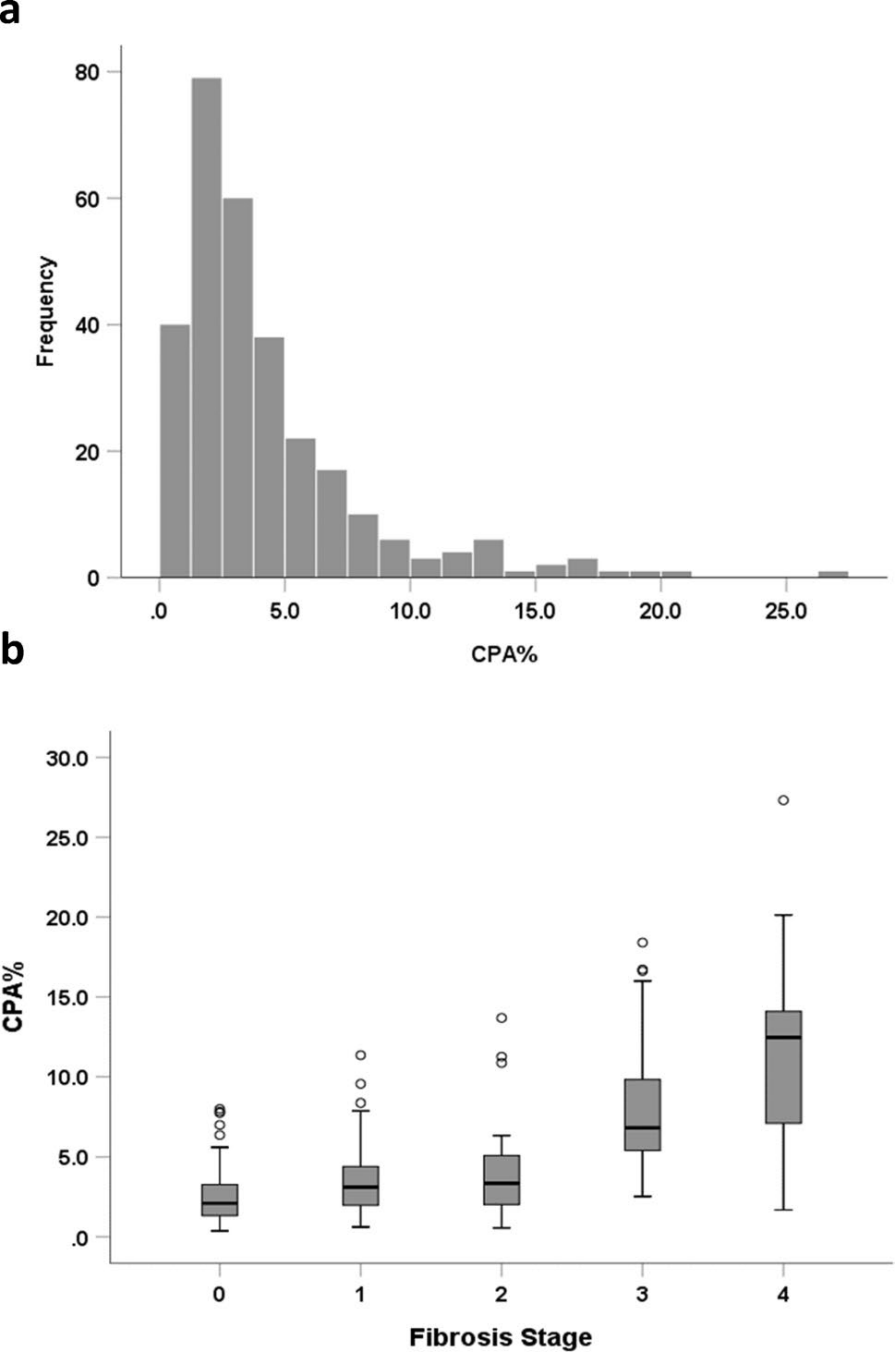
CPA distribution and interquartile ranges in each fibrosis stages. **a** CPA value distribution; **b** CPA values and pathologist determined fibrosis stages outliers greater than three times the SD range

The CPA values were skewed (demonstrated in Fig. 1a) with a median of 3.10% (range 0.40–27.3%). Nearly half of the biopsies had less than 3% of positive collagen staining area (141/295) and only 23 of them had CPA higher than 10%. The median and interquartile range of CPA values across fibrosis stages were 2.11 [1.32–3.31] for no fibrosis, 3.11 [1.93–4.41] for F1, 3.35 [1.89–5.23] for F2, 6.83 [5.30–9.87] for F3, and 12.46 [6.95–14.85] for F4. A significant correlation was demonstrated between CPA values and histopathologist-determined fibrosis stage with a Spearman correlation co-efficient of 0.549 (p < 0.001; Fig. 1b).

### CPA and long-term clinical outcomes

Over the study follow-up period (median 9 [0.2–25] years), totalling 3,253 person-years, there were 40 deaths, among which 11 of them died from liver causes. Liver decompensation occurred in 14 participants, HCC in 7, and combined liver outcomes occurred in 17. Overall survival in the cohort was 74%, with freedom from liver decompensation 94%, survival from liver death 95%, and freedom from HCC was 97%.

**Fig. 2:**
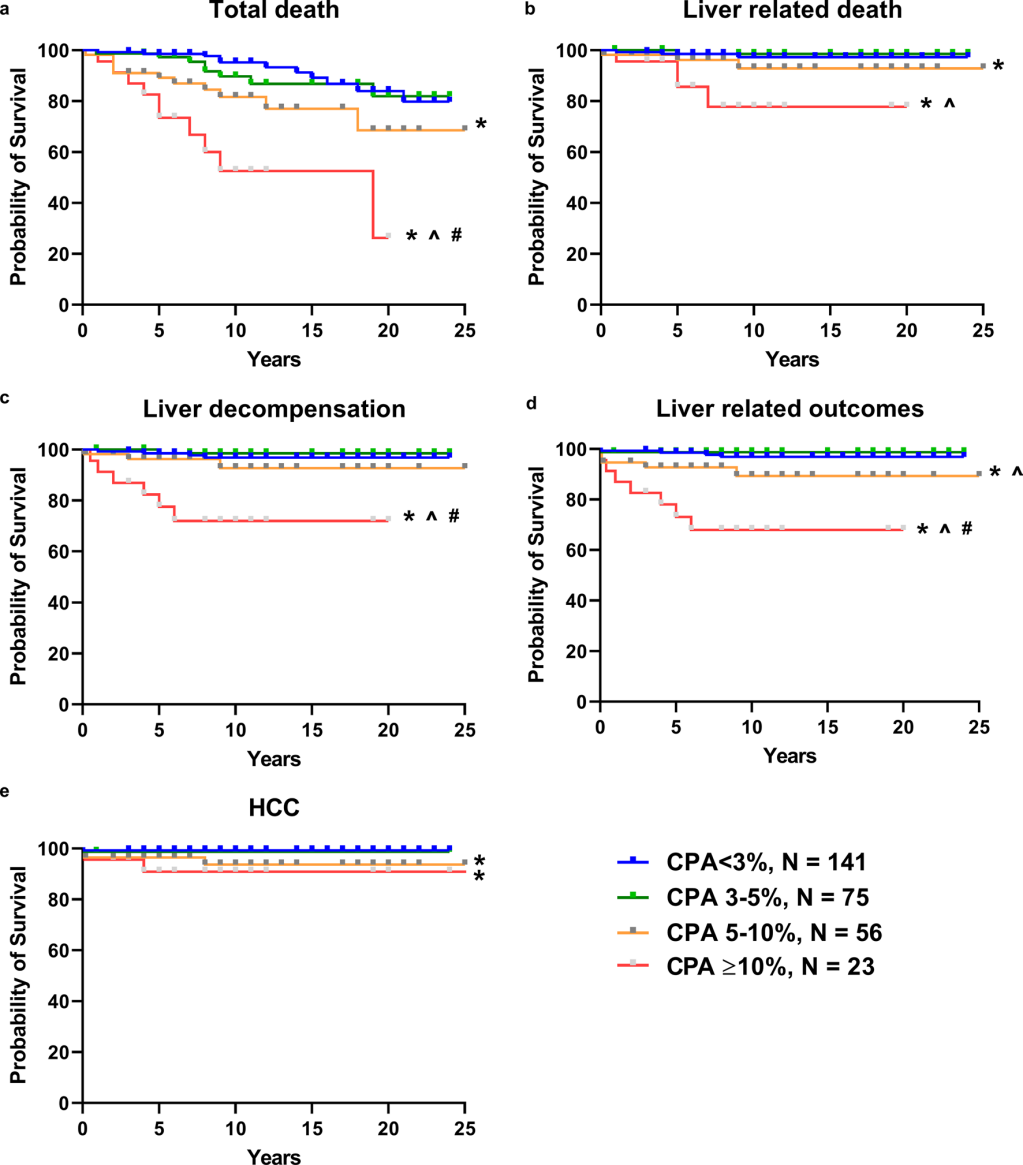
Long-term outcome risks in patients with biopsy proven NAFLD with different levels of CPA values; **a** all-cause mortality; **b** liver-related mortality; **c** liver decompensation; **d** HCC; **e** liver-related outcomes; *log-rank *p* < 0.05 vs. CPA < 3%; ^*p* < 0.05 vs. CPA 3–5%; ^#^*p* < 0.05 vs. CPA 5–10%

CPA values were significantly associated with higher risk for total death, LRD, liver decompensation, HCC as well as combined liver-related outcomes by Kaplan–Meier survival curve analysis (Fig. 2). Participants who had ≥ 10% CPA had the highest risk for all outcomes compared to those who had the lowest CPA (all p < 0.05). Patients with CPA 5–10% vs. CPA < 3% had a higher risk of adverse outcomes with the exception of liver decompensation (all p < 0.05). Cox regression modeling demonstrated similar results (Table 2). CPA > 10% was associated with a nine fold increased risk in death [Hazard ratio (IQR) 9.01 (3.78–21.46)], approximately tenfold increased risk of LRD (9.90 [2.21–44.43]) and 13-fold higher risk of combined liver outcomes (13.04 [3.80–44.73]). The results remained similar after multi-variable adjustment for age, diabetes, and hypertension (Table 2).

**Table 2:** Cox regression of CPA in predicting long-term clinical outcomes

	CPA < 3% (*N* = 141)	CPA 3–5% (*N* = 75)	CPA 5–10% (*N* = 56)	CPA ≥ 10% (*N* = 23)	*p* for trend
Total mortality					
Univariable	Reference	1.35 [0.54–3.36]	2.98 [1.29–6.89]	9.01 [3.78–21.46]	*p* < 0.001
Multivariable*	Reference	1.50 [0.55–2.33]	2.14 [0.85–5.43]	4.97 [1.87–13.22]	*p* = 0.013
Liver-related death					
Univariable	Reference	0.63 [0.07–6.07]	2.74 [0.55–13.60]	9.90 [2.21–44.43]	*p* = 0.004
Multivariable*	Reference	1.80 [0.11–29.03]	6.44 [0.65–63.66]	18.96 [1.98–181.96]	*p* = 0.034
Combined liver outcomes					
Univariable	Reference	0.48 [0.05–4.31]	3.50 [0.94–13.05]	13.04 [3.80–44.73]	*p* < 0.001
Multivariable*	Reference	0.96 [0.09–10.62]	4.85 [0.91–25.82]	15.61 [3.10–78.55]	*p* = 0.002

Data were presented as hazard ratios and 95% confidence intervals

*Multivariable adjustment included age, diabetes, and hypertension. Combined liver outcomes was defined as liver decompensation, HCC, or liver-related death, whichever occurred first

When analyzing CPA as a continuous variable, similar results were demonstrated with HR of 1.18 (IQR 1.07–1.29) in the multi-variable adjusted model for LRD. Similar trend was also demonstrated in the risk of total mortality (1.14 [1.07–1.22]) and combined liver outcomes (1.16 [1.08–1.26]). Stratifying the cohort into quartiles of CPA demonstrated that only those in the highest quartile (CPA ≥ 5.4%) had increased risk of outcomes (Supplementary Table 1).

### Prognostic accuracy of CPA in comparison to pathologist fibrosis stage and serum fibrosis markers

The accuracy of CPA, fibrosis stage, and NITs to predict outcomes as determined by AUROC is listed in Table 3. CPA had modest capacity in predicting total mortality (0.675 [0.577–0.773]), LRD (0.720 [0.558–0.883]), and combined liver outcomes (0.747 [0.615–0.879]), and was comparable to pathologist fibrosis stage (total mortality 0.701 [0.604–0.798]; LRD 0.770 [0.595–0.945]; combined liver outcomes 0.780 [0.644–0.915]). Hepascore had higher AUROC values compared to CPA, however, it did not reach statistical significance (LRD 0.911 vs. 0.720, *p* = 0.091; combined liver outcomes 0.870 vs. 0.747, *p* = 0.204) except for total death (0.860 vs. 0.675, *p* = 0.009). Similar accuracy was demonstrated between CPA, APRI, and FIB4 (Table 3).

**Table 3:** Accuracy of CPA, fibrosis stage, and non-invasive serum markers to predict outcomes using area under the ROC curve

	Total mortality	*p*	Liver-related death	*p*	Combined liver outcome	*p*
CPA (continuous)	0.675 [0.577–0.773]	–	0.720 [0.558–0.883]	–	0.747 [0.615–0.879]	–
Fibrosis stage	0.701 [0.604–0.798]	0.707	0.770 [0.595–0.945]	0.683	0.780 [0.644–0.915]	0.734
Hepascore	0.860 [0.763–0.956]	**0.009**	0.911 [0.849–0.973]	0.091	0.870 [0.748–0.992]	0.204
APRI	0.700 [0.575–0.826]	0.756	0.832 [0.690–0.973]	0.374	0.774 [0.627–0.921]	0.800
FIB4	0.818 [0.724–0.912]	0.053	0.845 [0.693–0.997]	0.315	0.864 [0.756–0.972]	0.231

Data were presented as area under the ROC curve (AUROCs) and 95% confidence interval. *p* value was calculated by two independent AUROCs’ comparisons

Bolded text is considered statistically significant

## Discussion

This study demonstrated the capacity of semi-automatic CPA quantification of liver biopsy to predict long-term clinical outcomes in patients with NAFLD. Higher CPA values were significantly associated with increased risks in total mortality, liver-related death, and overall liver outcomes including decompensation and occurrence of HCC. Notably, the capacity of CPA to predict clinical outcomes was comparable to pathologist determined fibrosis stage and non-invasive serum markers.

The association between higher CPA and poorer liver outcomes has previously been demonstrated in patients with other etiologies of chronic liver diseases. CPA was first shown to be predictive of liver decompensation in patients with chronic hepatitis C after transplantation [20]. Later, the risk stratification of CPA capacity was extended to liver-related death and HCC [12]. CPA has also been shown to predict risks of disease-related mortality in patients with primary sclerosing cholangitis [21]. Our current study provides further evidence of the correlation between CPA and risks of long-term clinical outcomes, which is comparable to the previous studies involving patients with alcohol and non-alcoholic hepatitis. Israelsen et al*.* showed that CPA predicts liver-related death and liver decompensation in patients with alcoholic hepatitis [22]. Buzzetti et al*.* also confirmed that CPA values from 10 × magnification predicted risks of LRD with similar effect size in 437 patients with biopsy proven NAFLD [23]. However, they only used the continuous CPA in the model and the effect size of CPA was small with 1% increase in CPA value increased the risk of LRD by 4% to 8% (hazard ratio 1.04–1.08). In our study, the hazard ratio for continuous CPA was 1.18 (IQR 1.07–1.29) for LRD prediction. Our study also demonstrated the increased risks for adverse outcomes only became significant when CPA exceeded 10% compared to patients with CPA < 3%. A high degree of accuracy of CPA for predicting hepatic decompensation has been reported for patients with cirrhosis due to chronic hepatitis C (AUC = 0.82–0.91) [9, 10]. The apparent higher accuracy is likely related to spectrum bias as only cirrhosis patients were included in these studies. It is also possible that CPA may be less accurate in quantifying finer ‘chicken-wire’ type fibrosis typically observed in NAFLD and ALD, in comparison to the predominately portal based fibrosis in chronic hepatitis C infection.

Consistent with previous groups, we identified a suboptimal capacity of CPA in predicting clinical outcomes with AUROC less than 0.8 [22, 23]. In our study, the AUROCs of non-invasive fibrosis tests for liver outcomes were higher, although not significantly, than CPA and fibrosis staging, especially for Hepascore and FIB-4. CPA is non-subjective and semi-automatic with excellent intra-observer concordance in this study (correlation co-efficient 0.99) and excellent inter-observer concordance demonstrated by others [23, 24]. Nonetheless, it is still susceptible to the potential biases from the liver biopsy, i.e., sampling error [25] which is avoided by serum NITs. However, further studies are required to confirm NITs being superior to liver histology in predicting long-term outcomes.

Additional automated methods for quantifying liver fibrosis beyond CPA have been examined including dual-photon microscopy which allows quantification of the textural features of collagen fibres, and machine learning approaches that capture the severity and heterogeneity of fibrosis [26, 27]. These approaches have also been demonstrated to predict liver outcomes; however, further work is required to determine if they have superior prognostic capability compared to CPA or NITs.

The limitations of this study included the cohort collection from a single center with a relatively small sample size and limited number of outcomes despite a relatively long follow-up period with a median of 9 years. Although no patients were on treatments at baseline which might alter their natural history, such as vitamin E, pioglitazone, or a glucagon-like peptide-1 receptor agonist, we cannot exclude that these may have been prescribed during follow-up. Nonetheless, comparative analyses between CPA, liver histology, and NITs remain valid. Also, nearly half the cohort did not have liver fibrosis which might impact the significance of the result; however, this strengthens the generalizability of our findings to populations with relatively low prevalence of advanced fibrosis.

In conclusion, this study confirms that the CPA stained by Sirius Red and analyzed by semi-automatic quantification predicts long-term clinical outcomes including total mortality, liver-related death, liver decompensation, and hepatocellular carcinoma in patients with biopsy proven NAFLD. The accuracy of CPA in prediction of outcomes was comparable to pathologist fibrosis staging and non-invasive serum markers including Hepascore, FIB-4, and APRI. Our data support serum NITs to be equivalent and possibly superior to CPA in the prediction of outcomes in NAFLD patients and re-inforces their utility in clinical practice.

### Supplementary Information

Below is the link to the electronic supplementary material.

Supplementary file1 (TIFF 827 KB)

Supplementary file2 (TIFF 19036 KB)

## Data Availability

Not applicable.
