# Supplementary figures and images for: Liver fibrosis quantified by image morphometry predicts clinical outcomes in patients with non-alcoholic fatty liver disease
Source: Hepatol Int. 2023 Jun 26;17(5):1162–9. doi: 10.1007/s12072-023-10564-3 (PMC10522738; doi:10.1007/s12072-023-10564-3)

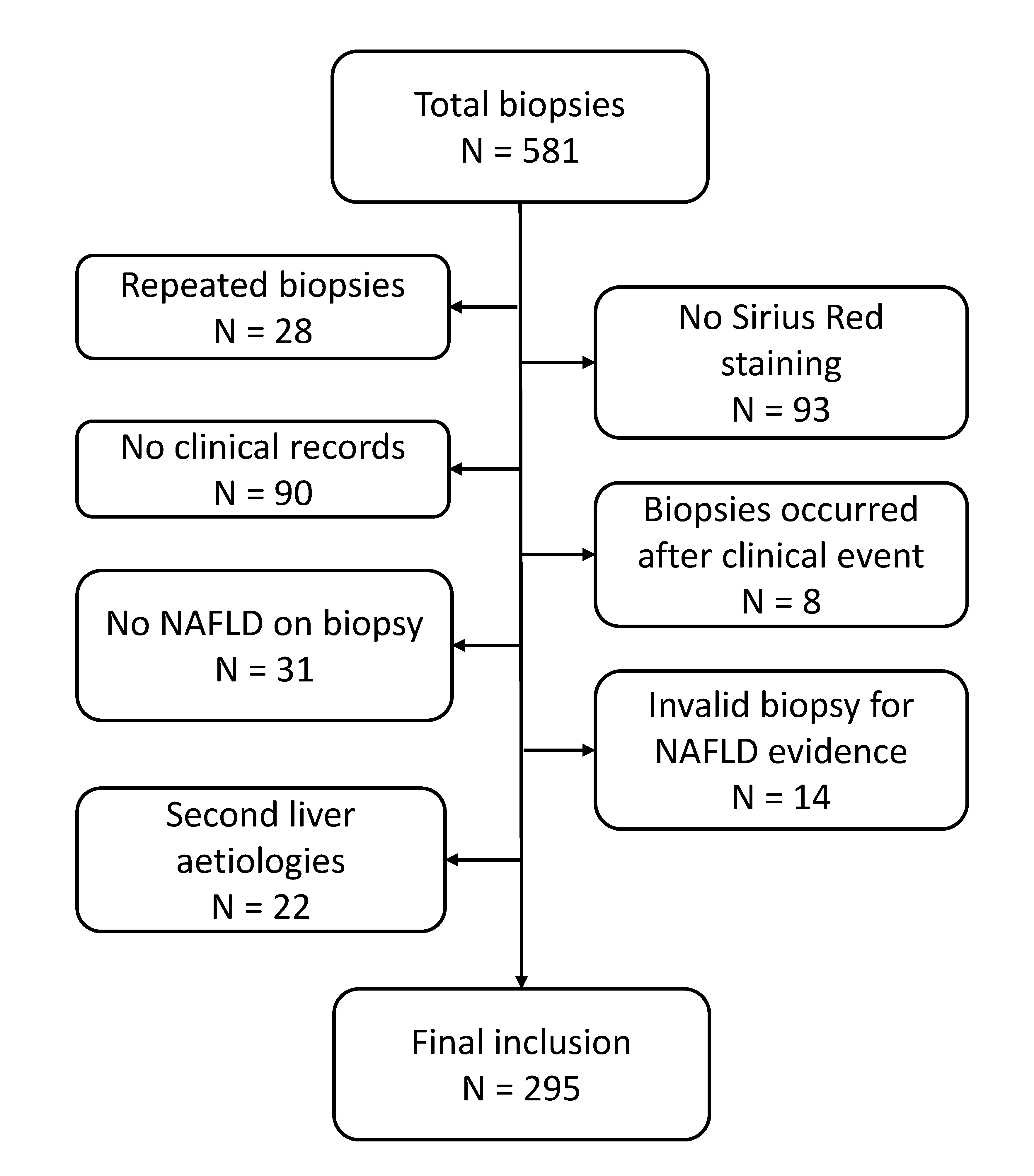

Supplement: Supplementary file 1 — Supplementary file1 (TIFF 827 KB) [file 12072_2023_10564_MOESM1_ESM.tiff]

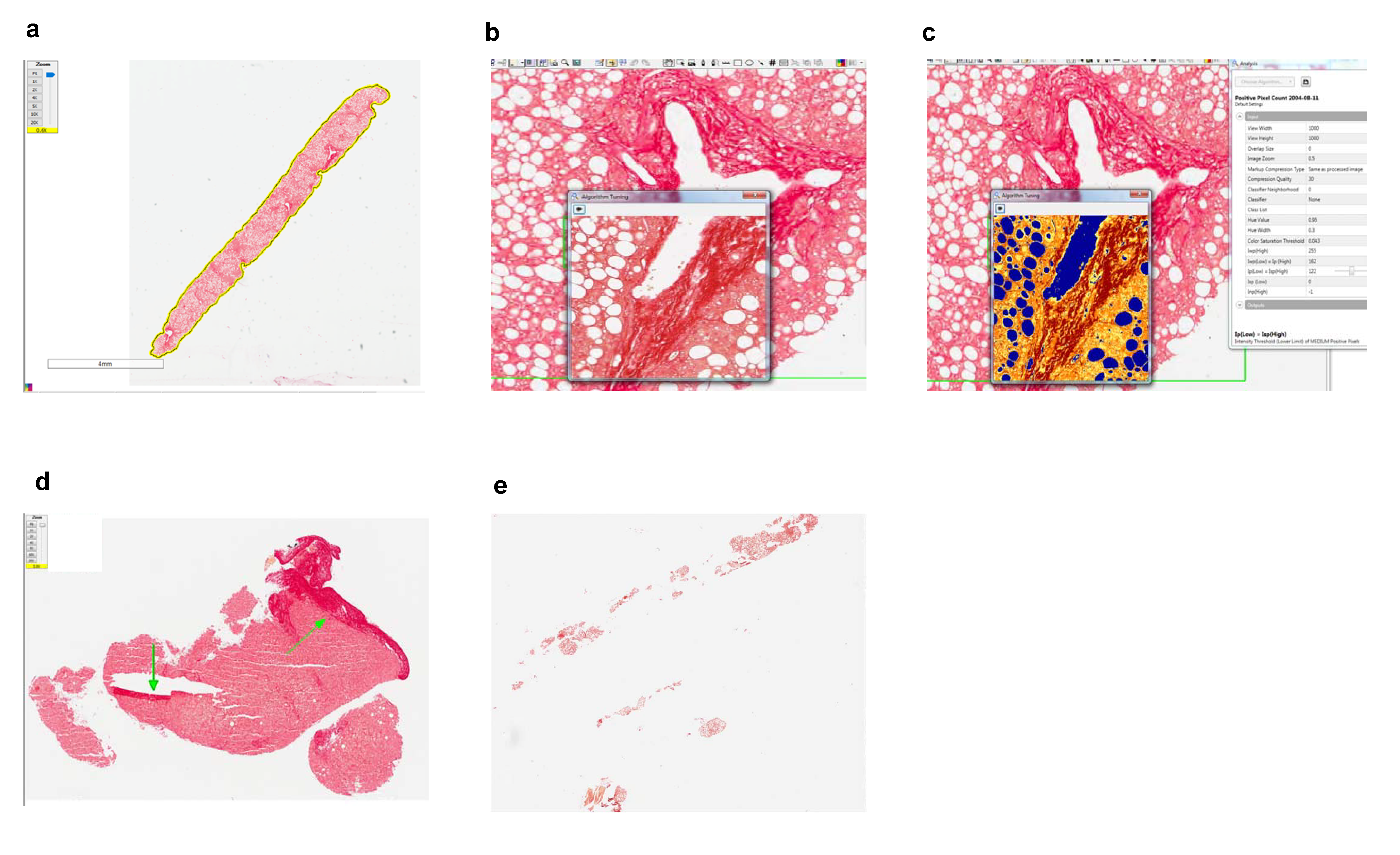

Supplement: Supplementary file 2 — Supplementary file2 (TIFF 19036 KB) [file 12072_2023_10564_MOESM2_ESM.tiff]
